# Quantum Chemistry‐based Molecular Dynamics Simulations as a Tool for the Assignment of ESI‐MS/MS Spectra of Drug Molecules

**DOI:** 10.1002/chem.202200318

**Published:** 2022-04-01

**Authors:** Romina Schnegotzki, Jeroen Koopman, Stefan Grimme, Roderich D. Süssmuth

**Affiliations:** ^1^ Institut für Chemie Technische Universität Berlin Straße des 17. Juni 124 10623 Berlin Germany; ^2^ Mulliken Center for Theoretical Chemistry Institute for Physical and Theoretical Chemistry University of Bonn Beringstr. 4 53115 Bonn Germany

**Keywords:** mass spectrometry, structure elucidation, fragmentation pathway, collision induced dissociation, quantum chemistry

## Abstract

In organic mass spectrometry, fragment ions provide important information on the analyte as a central part of its structure elucidation. With increasing molecular size and possible protonation sites, the potential energy surface (PES) of the analyte can become very complex, which results in a large number of possible fragmentation patterns. Quantum chemical (QC) calculations can help here, enabling the fast calculation of the PES and thus enhancing the mass spectrometry‐based structure elucidation processes. In this work, the previously unknown fragmentation pathways of the two drug molecules Nateglinide (45 atoms) and Zopiclone (51 atoms) were investigated using a combination of generic formalisms and calculations conducted with the Quantum Chemical Mass Spectrometry (QCxMS) program. The computations of the *de novo* fragment spectra were conducted with the semi‐empirical GFNn‐xTB (n=1, 2) methods and compared against Orbitrap measured electrospray ionization (ESI) spectra in positive ion mode. It was found that the unbiased QC calculations are particularly suitable to predict non‐evident fragment ion structures, sometimes contrasting the accepted generic formulation of fragment ion structures from electron migration rules, where the “true” ion fragment structures are approximated. For the first time, all fragment and intermediate structures of these large‐sized molecules could be elucidated completely and routinely using this merger of methods, finding new undocumented mechanisms, that are not considered in common rules published so far. Given the importance of ESI for medicinal chemistry, pharmacokinetics, and metabolomics, this approach can significantly enhance the mass spectrometry‐based structure elucidation processes and contribute to the understanding of previously unknown fragmentation pathways.

## Graphical abstract

**The previously unknown** fragmentation pathways of the two drug molecules Nateglinide (45 atoms) and Zopiclone (51 atoms) are completely elucidated using a combination of generic formalisms and calculations conducted with the Quantum Chemical Mass Spectrometry (QCxMS) program. The computations of the *de novo* fragment spectra were conducted with the semi‐empirical GFNn‐xTB (n=1, 2) methods and compared against Orbitrap measured electrospray ionization (ESI) spectra in positive ion mode.

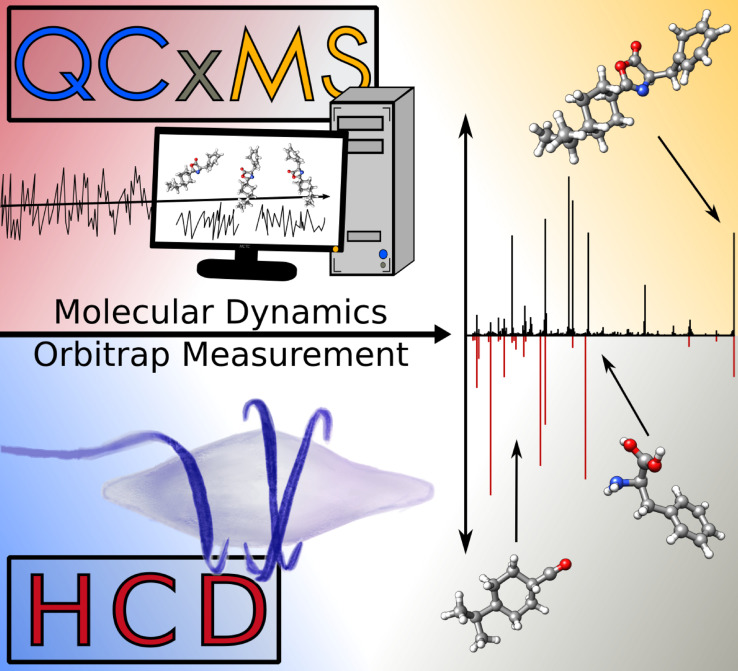

## Introduction

Mass spectrometry (MS) is a highly sensitive core analytical technique for researchers of various disciplines ranging from organic chemistry, medicinal chemistry to biochemistry and includes a vast amount of pharmaceutical, environmental, and forensic applications. The central analytical parameters are the structure and the amount of the analyte, reflecting qualitative and quantitative aspects of the analytics. Key to the fragmentation process is the nature of the analyte and the ionization technique applied.

Electron ionization (EI) is an ionization technique applied to volatile, preferably non‐polar analytes. The process of the formation of the odd‐electron (OE) radical cations [M]^.+^ (with commonly 70 eV kinetic energy of the bombarding electron) leads to the subsequent fragmentation of the molecule in a highly reproducible manner.[^1^, ^2^, ^3^] Hence, virtually classical fragmentation rules have been derived^[4]^ and the fragment‐rich mass spectra commonly are deposited in databases for straightforward compound identification.^[5]^

Over the past decades, electrospray ionization (ESI) combined with collision induced dissociation (CID)^[6]^ has evolved into the most commonly applied analytical ionization technique in medicinal chemistry and pharmacokinetics/pharmacodynamics (PK/PD) applications.[^7^, ^8^] This can be attributed to the predominantly polar nature of the analytes, which makes ESI highly complementary to EI.

The fragment assignment in the measured spectra and the resulting structure elucidation is commonly based on empirical rules, in which the (de‐)protonated molecule is subsequently fragmented either by charge‐migration (CMF) or charge retention fragmentation (CRF).[^9^, ^10^, ^11^, ^12^] However, while these rules can yield satisfying fragment ion assignments, fragmentation patterns are often observed that cannot straightforwardly be explained by CMF or CRF.[^9^, ^13^, ^14^] Furthermore, competing fragmentation pathways can increase the level of difficulty for describing the “real” fragmentation process, leading to uncertainty about the correct spectral assignment.[^15^, ^16^] In earlier studies, molecule fragmentation mechanisms of [M+H]^+^ ions were investigated either based on literature and personal experience, rather than experimental evidence.[^10^, ^17^, ^18^] Due to the associated uncertainties resulting from using the generic rules, theoretical methods have to be developed that are able to support the interpretation of ESI‐tandem mass spectrometry (MS/MS) fragmentation.

To date, it has become computationally affordable to use quantum chemical (QC) methods to calculate mass spectra. Most recently, quantum mechanical calculations found their way into the prediction of EI fragment spectra.^[19]^ The structural assignment of fragment ions is based on molecular dynamics (MD) simulations in which the fragmentation of the molecular ion is calculated along multiple, cascading trajectories. Unlike to already established computational approaches,[^20^, ^21^, ^22^, ^23^, ^24^, ^25^, ^26^] the on‐the‐fly quantum chemical calculation of the potential energy surface (PES) enables an unbiased determination of the composition of fragments and intermediate structures and does not depend on already known fragmentation mechanisms or database spectra.[^19^, ^27^, ^28^, ^29^, ^30^, ^31^, ^32^]

The usage of MDs to simulate collision induced dissociation (CID) reactions has also been investigated in other contributions.[^15^, ^16^, ^33^, ^34^, ^35^, ^36^] However, no direct comparison between measured and calculated signals was conducted, so that an overall agreement between experiment and theory could not be illustrated. Furthermore, the systems under consideration were rather small (size <20 atoms; molecular mass <170 Da), so that their complexity does not represent most common drug or macro‐molecules.

In this report, the mass spectra of the two drug molecules nateglinide and zopiclone were investigated using the **q**uantum **c**hemical **m**ass **s**pectrometry program QCxMS (x=EI, CID) in positive ion CID mode. The calculated results were compared to experimental measurements produced with an Orbitrap Fusion ESI‐MS/MS instrument leading to the complete fragmentation schemes of both drug molecules. In earlier work with QCxMS and its predecessor QCEIMS, it was shown that semi‐empirical quantum mechanical (SQM) methods, especially the GFN1‐xTB^[37]^ and GFN2‐xTB^[38]^ methods, can successfully be applied to calculate theoretical mass spectra, that agree reasonably well with database spectra.[^32^, ^39^] Using the automatic computation of fragment ion structures demonstrates the potential of QC calculations to become an important standard in matching experimental data and how to use this tool for fragmentation pathway interpretation.

## Methodology

In the following considerations, the protonated molecules [M+H]^+^ selected for collision‐induced fragmentation are referred to as precursor ions and their fragments as product ions. For electrospray ionization (ESI), relative low internal (thermal excitation) energies are utilized, usually leading to ions with paired electrons referred to as even‐electron (EE) or closed‐shell ions. The resulting precursor ion is commonly fragmented in CID experiments.^[6]^ On that account, the ionization and activation processes have to be treated separately. For the interpretation of the fragmentation routes, bond fissions are categorized as homolytic and heterolytic. In ESI‐MS/MS, heterolytic cleavage is observed almost exclusively.^[40]^ The charge either remains on the initial atom or is transferred to the cleaved fragment, respectively.^[41]^

### Benchmark molecules

Two different drug classes were considered: the hypoglycemic agent nateglinide and the sedative zopiclone. Nateglinide (M=317.4 Da, C_19_H_27_NO_3_) is an anti‐diabetic drug from the class of hypoglycemic agents, which lowers the glucose levels in the blood for the treatment of Diabetes mellitus. Zopiclone (M=388.1 Da, C_17_H_18_ClN_6_O_3_) acts as an agonist for the neurotransmitter gamma‐aminobutyric acid (GABA) receptor and works as a sedative. Both molecules differ in size and contain functional groups, which are representative of various drug molecules. Given that for the compound identification the existence of signals is of greater importance than the relative and absolute ion intensities, this work solely focused on comparing signals that exist in both, calculated and measured spectra, rather than the discussion of ion counts or each discrepancy between the theory and experiment. Details on the possible reasons for the diverging of some calculated signals are discussed later.

### Experimental details

All MS experiments were performed using an Orbitrap Fusion ETD mass spectrometer coupled to an UltiMate 3000 RSLC nano LC system (Thermo Fisher Scientific GmbH, Bremen, Germany). The samples were ionized using an electrospray needle with a voltage of 3800 V (ES+) and a sheath gas pressure of 4 Arb. The vaporizer temperature was adjusted to 35 °C. The precursor ions were mass‐selected using a linear ion trap and allowed to collide in an HCD collision cell with N_2_ in a stepped collision energy mode at HCD collision energies of 15, 60, 120 %. The isolation width was set to *m/z* 0.7 with an AGC target of 2.0^4^ and a resolution of 60,000. Based on the S/N ratio, only counted fragments with a relative intensity >1.5 % were considered. The measured compounds were isolated from film‐coated tablets (Zopiclon‐ratiopharm, Starlix Novartis) by crushing the solid and subsequent extraction with methanol for nateglinide and chloroform for zopiclone.

### Ranking of difficulty by common fragmentation pathways

**Scheme 1:**
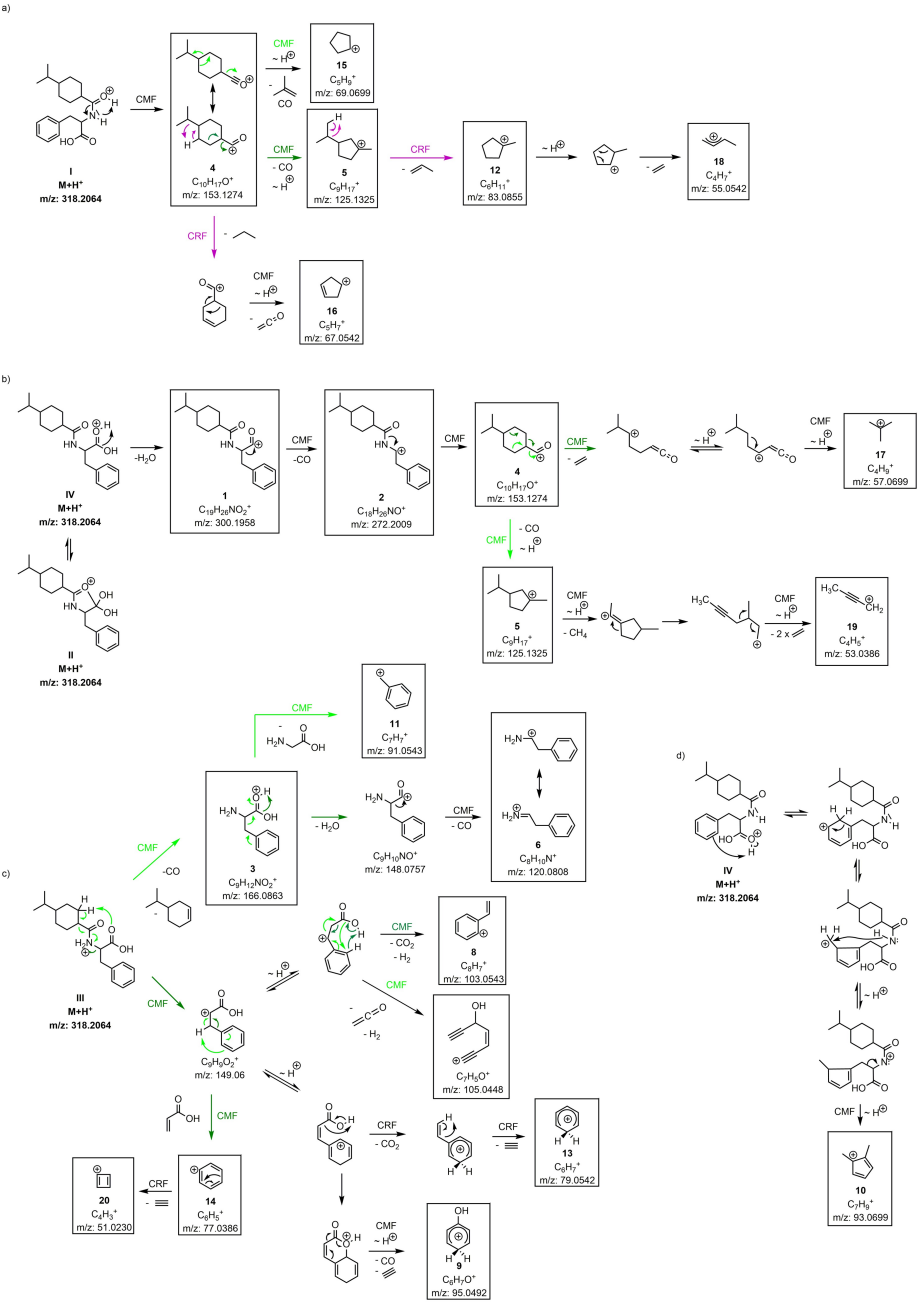
Proposed fragmentation pathways of nateglinide. Fragmentation according to a one‐channel fragmentation (black arrows) or branched fragmentation pathways (highlighted in coloured arrows; CMF green, CRF purple). *∼* H^+^ means proton migration. Boxed fragments were experimentally detected. Unboxed structures are “snapshots” on calculated trajectories and are displayed for clearer retracing of the reaction pathways; they are not global minima on the potential energy surface. **For matters of clarity, the neutral fragment has not been depicted in all cases**.

**Scheme 2:**
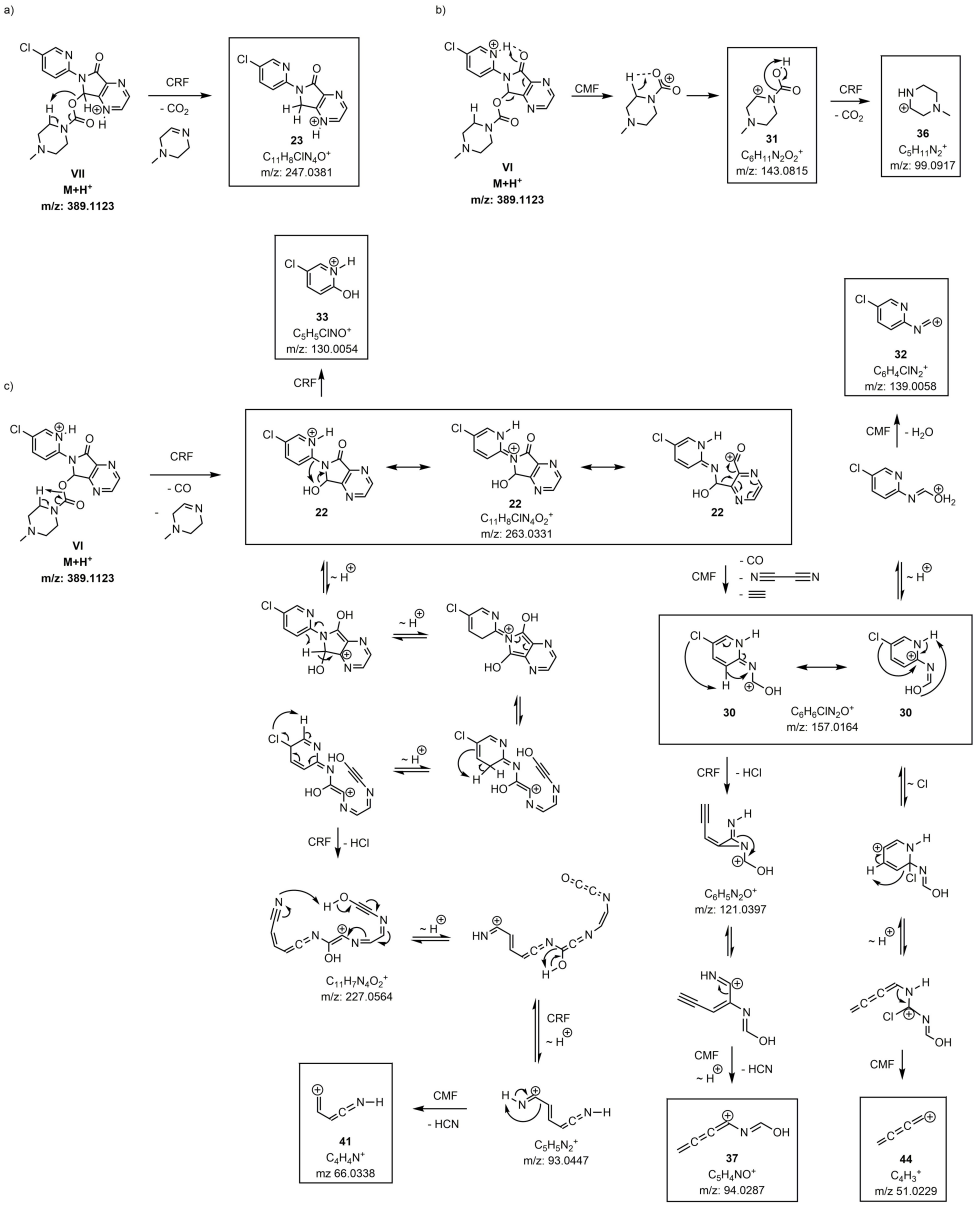
Proposed fragmentation pathways of zopiclone – part 1. Fragmentation according to a one‐channel fragmentation (black arrows) or branched fragmentation pathways (highlighted in coloured arrows; CMF green, CRF purple). *∼* H^+^ means proton migration. Boxed fragments were experimentally detected. Unboxed structures are “snapshots” on calculated trajectories and are displayed for clearer retracing of the reaction pathways; they are not global minima on the potential energy surface. **For matters of clarity, the neutral fragment has not been depicted in all cases**.

**Scheme 3:**
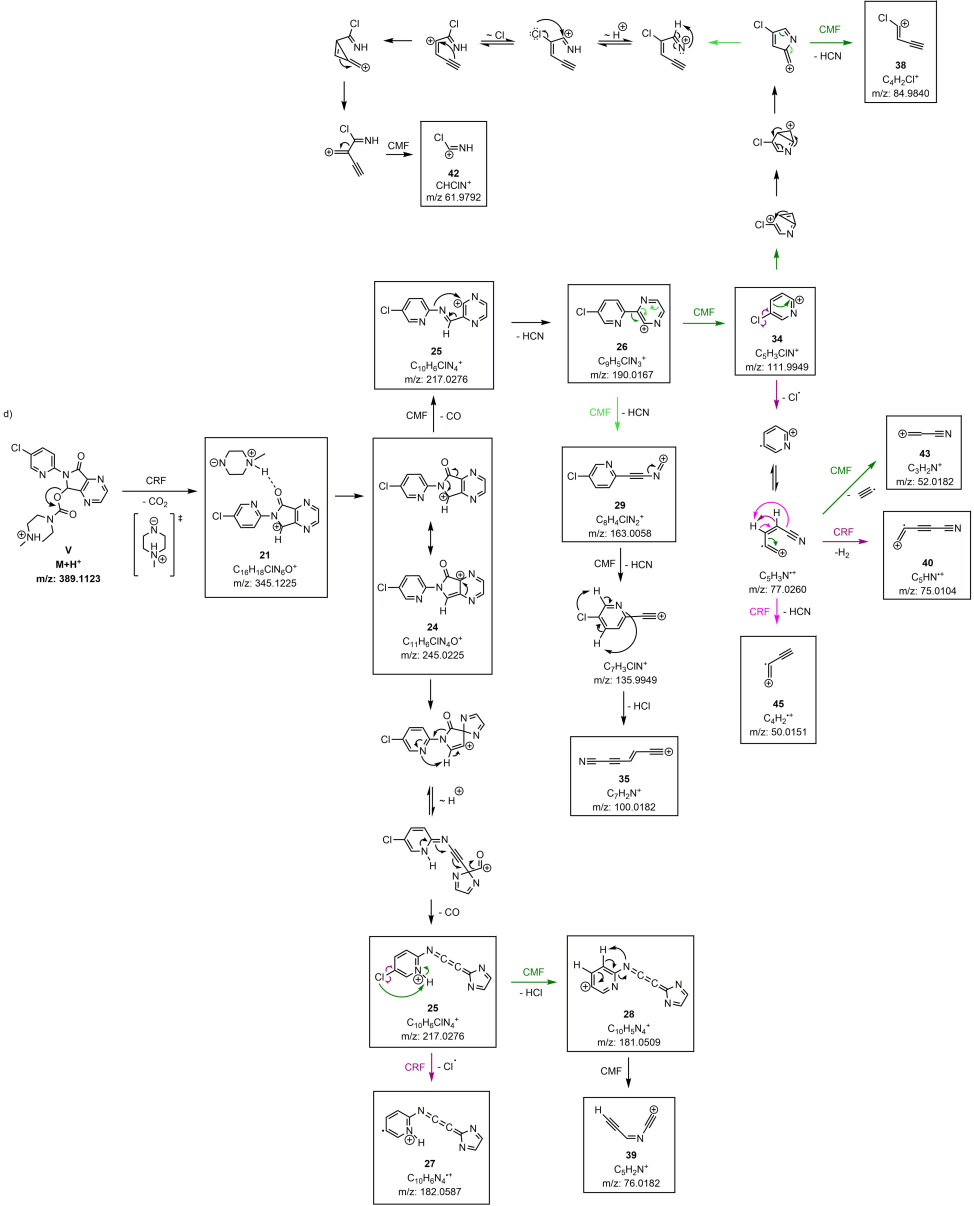
Proposed fragmentation pathways of zopiclone – part 2. Fragmentation according to a one‐channel fragmentation (black arrows) or branched fragmentation pathways (highlighted in coloured arrows; CMF green, CRF purple). *∼* H^+^ means proton migration. Boxed fragments were experimentally detected. Unboxed structures are “snapshots” on calculated trajectories and are displayed for clearer retracing of the reaction pathways; they are not global minima on the potential energy surface. **For matters of clarity, the neutral fragment has not been depicted in all cases**.

The degree of difficulty to describe the observed fragments was categorized by the feasibility to explain the shown pathways (Schemes 1–3) on rule‐based fragmentation descriptions known from literature^[9]^ (Table 1 & 2, column 4). Of the illustrated fragments, the experimentalist was able to infer those assigned as *feasible* with reasonable efforts based on the common rules. A comparison of the proposed fragmentation reactions to the simulated trajectories confirmed the described pathways. For fragmentation reactions with higher complexity, the elaboration of rearrangements and cleavages occurring in CID experiments can be very time consuming and requires vast experience. In that regard, the possibility to utilize theoretical trajectories was highly expedient, so these fragments were categorized as *with QCxMS*. In retrospect, we were then able to corroborate the compliance of these fragmentation pathways with the classical fragmentation rules. The third category contains fragments designated as *only QCxMS*. The fragmentation pathways could solely be described with the help of the simulated trajectories. Their rearrangements and cleavages are untypical and differ from the common rules in the literature.^[9]^

**Table 1:** Fragment list of nateglinide. Fragments **measured** by ESI‐MS/MS with intensity >1.5 % are listed according to their molecular mass. Fragments **simulated** by QCxMS are marked. Classification of the **rule‐based fragmentation** ranking as described before.

Fragment No.	Measured fragments	Simulated	Ruled‐based
	(m/z)		fragmentation
1	300.1962	yes	feasible
2	272.2011	yes	feasible
3	166.0862	yes	feasible
4	153.1274	yes	feasible
5	125.1325	yes	feasible
6	120.0808	yes	feasible
7	105.0448	yes	feasible
8	103.0543	yes	only QCxMS
9	95.0492	yes	with QCxMS
10	93.0700	yes	only QCxMS
11	91.0543	yes	feasible
12	83.0857	yes	feasible
13	79.0544	yes	feasible
14	77.0387	yes	feasible
15	69.0700	yes	feasible
16	67.0544	yes	feasible
17	57.0700	yes	feasible
18	55.0544	yes	feasible
19	53.0387	yes	with QCxMS
20	51.0231	yes	feasible

**Table 2:** Fragment list of zopiclone. Fragments **measured** by ESI‐MS/MS with intensity >1.5 % are listed according to their molecular mass. Fragments **simulated** by QCxMS are marked. Classification of the **rule‐based fragmentation** ranking as described before.

Fragment No.	Measured fragments	Simulated	Ruled‐based
	(m/z)		fragmentation
21	345.1227	yes	only QCxMS
22	263.0333	yes	feasible
23	247.0384	yes	feasible
24	245.0228	yes	feasible
25	217.0276	yes	feasible
26	190.0167	yes	feasible
27	182.0587	yes	with QCxMS
28	181.0509	yes	with QCxMS
29	163.0057	yes	feasible
30	157.0163	yes	with QCxMS
31	143.0815	yes	only QCxMS
32	139.0058	yes	feasible
33	130.0054	*no*	feasible
34	111.9949	yes	feasible
35	100.0183	yes	with QCxMS
36	99.0918	yes	feasible
37	94.0288	yes	with QCxMS
38	84.9841	yes	feasible
39	76.0183	yes	with QCxMS
40	75.0105	*no*	feasible
41	66.0340	yes	with QCxMS
42	61.9793	yes	with QCxMS
43	52.0183	yes	with QCxMS
44	51.0231	yes	with QCxMS
45	50.0152	yes	with QCxMS

### Computational details

Before the QCxMS simulations were conducted, protonation of the targeted species was achieved using the automated QC‐based protonation protocol^[42]^ of CREST^[43]^ at the GFN2‐xTB level of theory. The most populated protonated structures inside a 20 kcal/mol energy range were re‐optimized using density functional theory (DFT) at the PBEh‐3c level^[44]^ to guarantee the correct energy ranking. To provide the reader with a better understanding of the QCxMS protocol, the mechanics are discussed in short in the following. For an in‐depth discussion of the QCxMS program and its working mechanics, the reader is referred to the original publication.^[39]^

Basically, QCxMS runs in multiple steps.

1. **Ground state sampling**: the input structure is equilibrated at 600 K on a 15 ps MD trajectory with a timestep of 0.5 fs.
2. **Set‐up**: 1,250 structural snapshots were taken along a 30 ps MD trajectory for sampling of the conformational space.
3. **Production runs**: conducting massively parallel calculations with the snapshot structures as starting geometries.

In the production run step, the collisional activation is simulated. The collision simulations imply the following conditions for each starting geometry:

- rotation along the Euler axes to guarantee different impact sites.
- adding rotational energy of k_B_T/2 per principal axis, with k_*B*_ for the Boltzmann constant and T as the temperature of the ion.
- scaling of the internal energy prior to the collision along a 1 ps MD trajectory to standard distributed values between 4 and 8 eV.
- velocity scaling of [M+H]^+^ according to the acceleration potential of 10 eV E_COM_.

Subsequently, the collisions between [M+H]^+^ and neutral He gas atoms with a randomized collision angle (impact parameter *b*) are simulated for each product ion run. The collisions transform kinetic into the internal energy of the ion. If the critical energy E_0_ is reached, statistical and non‐statistical fragmentation of the molecular ion occurs. To induce sufficient dissociation of [M+H]^+^, the collision process had to be repeated multiple times.

Unfortunately, the exact number of collisions in the experiment cannot be determined. For the best reproduction of the measured spectrum, an automatic run‐mode was developed in QCxMS, that circumvents a tedious trial‐and‐error approach to determine the correct number of collisions and corresponding collision energies (see the original publication^[39]^). It uses the kinetic gas theory as an indication to solve this problem, in which the number of collisions can be calculated through the collision cell length, collision gas pressure and collisional cross‐section. For the calculations conducted in this work, the parameters were set to 1.25 cm cell length with 0.132 Pa collision gas pressure at a temperature of 300 K, which are in agreement with the program's default values. These values provide an estimate of the number of collisions, but do not affect any other simulation condition.

The calculations were conducted by QCxMS version 5.0 using the xTB version 5.8.1 on Intel Xeon E3‐1270@v5 3.6 GHz computer cores. The forces were calculated with GFN1‐xTB for zopiclone and GFN2‐xTB for nateglinide and applied with a timestep of 0.5 fs in the MD simulations. CREST version 2.8.1 using GFN2‐xTB was employed for the protonation of the molecule. Automatic re‐ranking was done by the ENSO^[45]^ script version 2.0.2. DFT calculations were performed using ORCA[^46^, ^47^, ^48^] version 4.2.1.

### Discrepancies between calculations and experiments

Some considerations have to be taken into account when comparing computed spectra with experimental measured results.

Depending on the design of the instrument used, the amount of internal energy of the precursor ion after ionization is commonly unknown. While efforts can be undertaken to determine this value, calibration and measurement need a lot of work and are thus not routinely conducted. The ionization source can influence the overall fragmentation process, depending on the “hardness” of the ionization, even sometimes leading to in‐source fragmentation. Further contributing factors involve details like the setup of the used quadrupoles, collision cell, and means of detection. Generalizability and reproducibility depend strongly on these factors, making a direct comparison even between different instruments complicated.

From the computational point of view, reasonable MD simulations run over shorter time scales (picoseconds) than experimental measurements (milliseconds). To conduct appropriate MD simulations on this time scale, semi‐empirical methods must be employed, sacrificing the accuracy of calculations for the sake of the computational cost. This can lead to the wrong description of the PES and thus false fragment structures and/or charge assignments. Furthermore, decomposition effects that occur after a long timescale can be missed by the simulations, while short‐lived fragments might be over‐represented.

In the current version of QCxMS, the precursor ions are accelerated once at the beginning of the simulation, so the loss of kinetic energy in multiple collisions is not compensated for, as it is done in modern MS instruments (here: Orbitrap Fusion). It was found that the agreement to measured spectra is increased drastically when subsequent collisions between created fragments and neutral gas atoms (fragment‐gas‐collisions (*fgc*)) were considered. Not accounting for re‐acceleration thus might lower the *fgc* collision energies in the calculations, leading to an underrepresentation of lower mass fragments.

## Results and Discussion

### Nateglinide

**Figure 1:**
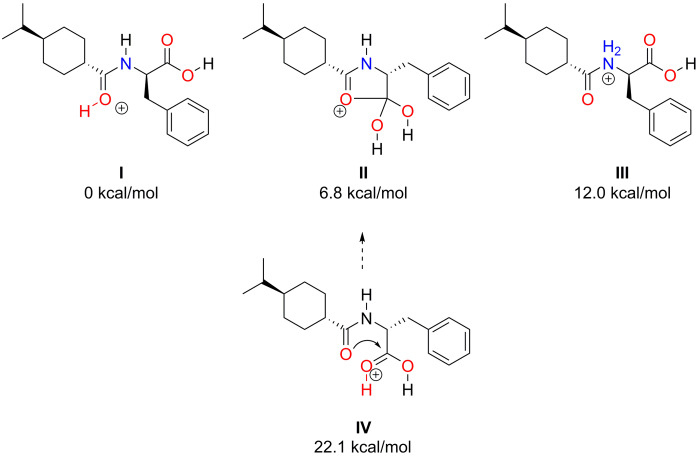
Protomers I–IV of nateglinide and their relative energies (kcal/mol) related to the protomer with the lowest protonation energy as obtained by PBEh‐3c DFT calculations.

The most populated protomers of nateglinide (M=317.4 Da, C_19_H_27_NO_3_) were determined using ENSO at 600 K. By the QCxMS calculations, it was found, that the protomers in the lowest 20 kcal/mol energy window contributed most to the final spectrum, while other protomer spectra did not provide additional information on the overall fragmentation behavior. The structures considered are displayed in Figure 1.

Using the classical protonation formalism, structures I and III can be created, while the structure of protomer II is non‐intuitive. The structure of protomer II was re‐optimized at PBEh‐3c and PBE0/def2‐TZVP‐D4 levels to gain further insight. It was found that after protonation of the carboxylic acid group, a ring formation occurred. Single point calculations at PW6B95‐D3/QZVPP level confirmed that the ring formation stabilizes this structure by 16.2 kcal/mol and is thus populated in the given energy window. However, by increasing the internal energy through collisions, higher energy structures can become populated and a ring‐opening occurs before the fragmentation takes place. Since the “mobile proton theory” gives strong evidence that protomers can easily rearrange into each other in the high‐temperature regime of mass spectrometry experiments, and several fragments originate from more than one protomer,[^49^, ^50^, ^51^] it is likely that the protomer structures displayed in Figure 1 can simultaneously be present in the measurements. Through the close vicinity of the heteroatoms in this structure, proton migration at high internal energy is promoted and the individually simulated spectra of the different protomers I, II, and III provided similar fragments with varying intensities (see Figure S1–3).

**Figure 2:**
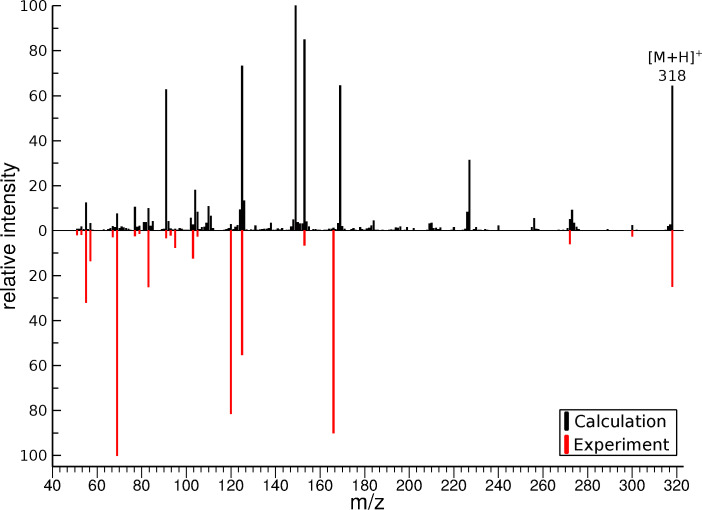
Comparison of the simulated ESI‐MS/MS spectrum computed by QCxMS@GFN2‐xTB with the experimental spectra (red, inverted) of nateglinide. The spectra for all three considered protomer structures are averaged with equal weight.

In Figure 2, the measured spectrum is compared to the calculated spectrum of nateglinide. The calculated spectrum is composed of the combined and normalized results from the calculations of all three protomer structures.

A detailed comparison of the signals showed that the measured fragments could be reproduced with 100 % agreement by the calculations. The corresponding complexities, as described earlier, are displayed in Table 1.

The comparison of the simulated spectra to the measured results display significantly more peaks, including differences in intensities. The additional peaks are no isotopologs. As mentioned before, differences between calculated and experimental spectra are to be expected. For the calculation of large molecules, a factor to consider is that the distribution of the collision energy into the ion's internal energy can take a long time due to the high number of degrees of freedom. Due to the shorter simulation times than reaction times and the use of SQM methods for the calculations, an underrepresentation of slower dissociation events and an overestimation in survival rates of non‐physical artifacts can be observed in the calculated spectrum. This also causes short‐lived fragments and intermediate structures in the simulations, leading to the small, unmatched signals in the theoretical spectrum. Using a higher level of QC theory, deficiencies in the PES calculation could be alleviated; however, this is currently not feasible for molecules of this size. Nevertheless, due to the fragment coverage of the calculation (Table 1), the molecule's fragment structures can be unequivocally assigned.

A formulation of a classical fragmentation scheme would start from the protomers formed by the protonation of heteroatoms bearing non‐binding electron pairs. The outlined fragmentation pathways for nateglinide are shown in Scheme 1. The CMF and CRF mechanisms of the three protomer structures (I–III) were drawn to retrace every step of fragment formation.

The description of the fragmentation routes for difficult fragments (*with QCxMS*) proved to be particularly challenging and heavily relied on the support of the calculated trajectories. For this reason, the fragmentation routes for the two fragments **8** (*m/z* 103) and **10** (*m/z* 93) could not be derived from common mechanisms published so far. With the help of the calculated trajectories, the untypical reaction pathways (*only QCxMS*) could now be elucidated. Ion **8** (*m/z* 103) undergoes a 1,6‐elimination of H_2_, resolving the aromaticity of the benzene ring (Scheme 1c). This reaction mechanism is not part of the classic fragmentation rules; mostly 2,4‐eliminations of H_2_ are observed. The trajectory can be found in the Supporting Information as “*elimination_reaction_ion8.mp3*”.

Fragment **10** (*m/z* 93) is obtained in the calculations via proton migration of protomer II from the protonated oxygen to the aromatic ring. Again, aromaticity is resolved, which according to generic rules is not favored and therefore would not be formulated (Scheme 1d). The corresponding trajectory can be found in the Supporting Information as “*elimination_reaction_ion10.mp3*”.

The breakdown of the aromatic compounds may be related to the high temperatures under which the reactions take place. The available energy makes reactions possible which, under “normal” circumstances, would have too high of a reaction barrier. These findings should be considered when establishing fragmentation patterns of aromatic compounds.

### Zopiclone

**Figure 3:**
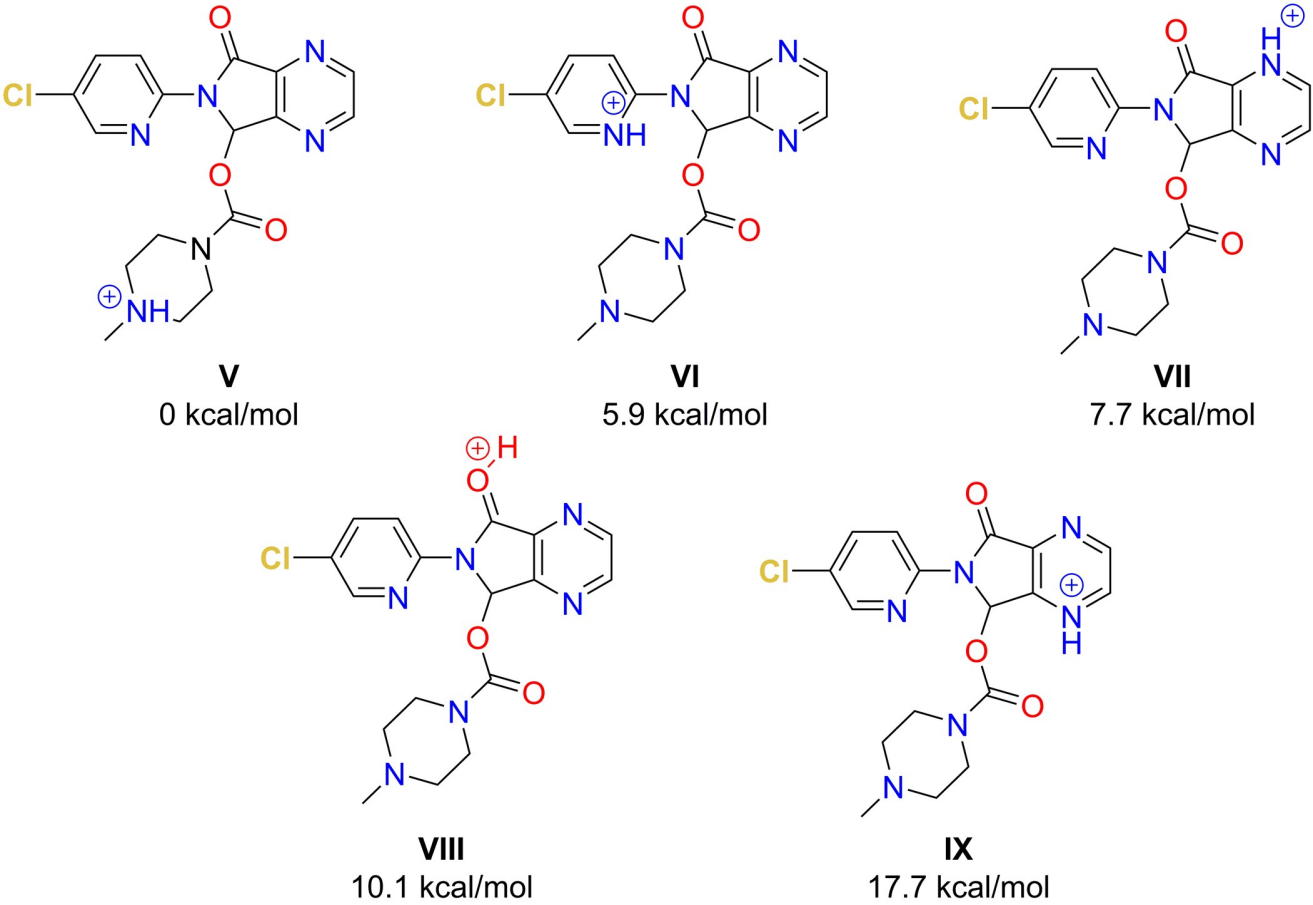
Protomers of zopiclone and their energies (kcal/mol) related to the protomer with the lowest protonation energy as obtained by PBEh‐3c DFT calculations.

For zopiclone (M=388.1 Da, C_17_H_18_ClN_6_O_3_), CREST computed five protomer structures that were populated in the 20 kcal/mol free energy range at 600 K (see Figure 3).

**Figure 4:**
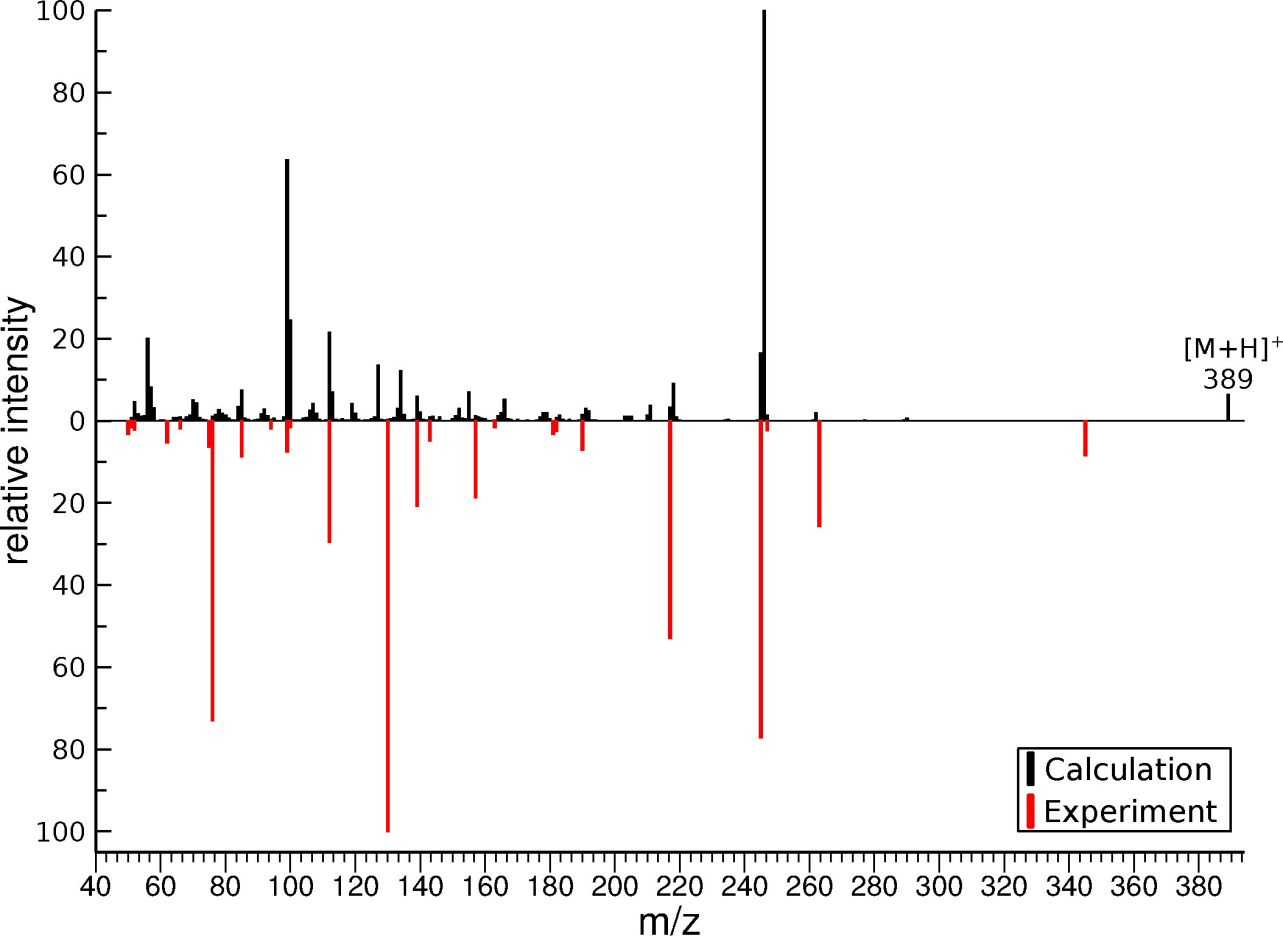
Comparison of the simulated ESI‐MS/MS spectrum computed by GFN1‐xTB (energy window 20 kcal/mol) with the experimental spectra (red, inverted) of zopiclone. The spectra for all five considered protomer structures are averaged with equal weight.

The combined spectrum of the protomers of zopiclone calculated with QCxMS is compared to the experimentally measured spectrum in Figure 4.

The QC calculations identified 23 out of the 25 fragments generated by the mass spectrometer, resulting in a coverage of the fragment pattern of 92 % (Table 2, column simulated). To illustrate the mechanistic details of the proposed fragmentation pathways for zopiclone, common fragmentation rules were used (Schemes 2 & 3).

Most of the observed fragments of zopiclone are formed by highly complex rearrangements and proton migrations (marked as *with QCxMS*, Table 2).

To determine a detailed illustration of these pathways by classical fragmentation schemes, considerable amounts of time and experience are required. These efforts extend even further if multiple protomer structures have to be considered. Due to the increased internal energy that the ions receive from the collision processes, the system is in a high‐temperature regime. With high internal energies, reaction barriers between the protomers can be exceeded which leads to proton mobility between the different starting structures. The exact population in this temperature regime is unknown and lacks detailed research, so it is not straightforward to weigh the influences of the protomers on the final spectrum based on free energies alone. Calculations on the different protomer structures revealed similarities in the main fragmentation behavior, thus it is to be expected that zopiclone creates a tautomeric network between the protonation sites. However, the simulated fragmentation pathways of protomer VI showed the best conformity with the generic rules, which allowed for the construction of the majority of fragmentation pathways based on this protomer. The CID of zopiclone showed two dissociations that do not undergo common CRF or CMF reactions[^6^, ^9^] (marked as *only QCxMS*, Table 2). The first example is complex **21**. It is formed by the ionic fragment and the zwitterionic piperazine fragment derived from the same precursor ion, which was stable enough to be detectable (*m/z* 345, Scheme 3d). Such ion‐dipole complex formations in the gas phase were described in several studies[^52^, ^53^, ^54^, ^55^, ^56^, ^57^] and occur when the energy threshold for the direct precursor decomposition is not reached. The complex formation enables reorientations, thereby allowing for transfers or reactions between parts of the molecule otherwise not possible due to the remoteness in the precursor molecule.^[54]^ The capability of QCxMS to indicate ion‐dipole complexes is of great value when interpreting and elucidating fragmentation pathways.

To confirm this finding, several independent MDs were calculated in which the two fragment units were placed close to each other and propagated over a 5 ps trajectory. The calculations were performed at a BLYP‐D3/def2‐SVP level at a temperature of 500 K. It was found that different H‐bond formations are possible – the actual formation depends strongly on the protonation site and the spatial arrangement of the reaction partners to one another at the start of the simulation. Calculations of the H‐bond binding energies might reveal the preferred binding sites and the binding situation in this context. Overall, the underlying formation of fragment **21** can be described using quantum‐mechanical principles, but due to the high number of possible binding situations and the extension of the underlying work, a detailed description of the exact binding situation is beyond the scope of this publication.

The second fragment formation addressed as *only QCxMS* (**31**, *m/z* 143, Scheme 2b) is another example indicating ion‐dipole complex formation. To describe the observed structure by common reaction mechanisms, a nucleophilic attack of a piperazine‐hydrogen on the oxygen of the carbamate group must occur, which results in a neutral cleavage of the main part of zopiclone. The attack of a hydride on a heteroatom, bearing already a negative partial charge, is contradictory. However, it can be resolved by looking into the simulated trajectories with their corresponding calculated energies, showing hydrogen transfers most likely induced by ion‐dipole interactions.

Remarkably, the theoretical calculations for fragments derived from **34** and **26**, predict the occurrence of the radical cations **45** and **27**, respectively. This seems plausible as the experimental occurrence of radicals has been described for heteroatoms of higher atomic numbers, e. g. SO_2_CH_3_ and Cl.^[40]^ It is important to notice, that the fragmentation pathway described in Scheme 2c is not sufficiently reproduced by the initial simulations. The relatively short simulation times and high collisional energies led to an under‐representation of the corresponding signals in the calculated spectrum. However, the proposed fragmentation pathway could be confirmed by using fragment **22** as starting structure for a separate QCxMS calculation. Here, all displayed dissociation events except for **33** (*m/z* 130, Scheme 2c) and **40** (*m/z* 75, Scheme 3) were described by the calculations and the resulting fragments could be generated sufficiently. The rearrangement reactions of fragment **33** and fragment **40** could not be calculated by the MD simulation. The formation of fragment **33** involves a substitution of a C−N bond with a C−OH bond, most probably involving an intermediate four‐ring formation between the neighboring C−N−C−O atoms. Most likely the underlying QC method might not be suitable to calculate this specific rearrangement reaction. Increasing the level of theory might solve this problem, but was not investigated in the course of this work. The same holds for fragment **40**, but in addition, the involved H rearrangement must succeed over a large distance, which reduces its possibility to occur in the simulations. Although fragments **33** and **40** could not be retraced by the simulations, the high congruence of the fragment patterns allows for a clear assignment.

## Conclusion and Outlook

In this work, the previously unknown collision‐induced fragmentation pathways of the two drug molecules nateglinide and zopiclone have been completely described by using a combination of generic formalisms and quantum chemical calculations conducted with the Quantum Chemical Mass Spectrometry (QCxMS) program in positive ion mode. The most populated protomers within a defined energy window of 20 kcal/mol were used as starting points to calculate corresponding fragment spectra, allowing for multiple reaction pathways. Utilizing molecular dynamics simulations, the MS/MS spectra could be calculated and compared to the experimental spectrum, achieving an excellent coverage >90 % of the measured signals. Fast and easy access to the calculated trajectories was an important complementation to the classical interpretation of the final fragmentation pathway, which substantially supported the experimentalist's interpretation of the CID spectra, while simultaneously reducing the time of structure elucidation significantly. Uncommon fragmentation pathways were identified which encourage further investigation and might lead to an expansion of what to consider as “typical fragmentation pathways”.

The investigated structures were of typical size for common bioactive molecules and contained functional groups which are representative for several drug classes. In that context, it is worth mentioning, that the complexity of the fragment spectra rapidly increases with the molecular mass and number of heteroatoms. Given the enormous structural diversity, e. g. of drug‐like synthetic molecules, a thorough interpretation of fragment spectra without the aid of QC calculations becomes unrealistic and is eventually based on improper assumptions. For the first time, the presented work showed how the automatisms implemented in QCxMS combined with the fast calculations conducted with GFN‐xTB can be used to completely elucidate molecules of this size and complexity. In summary, QCxMS proved to be a valuable tool to facilitate the detailed illustration of fragmentation mechanisms or even enables a description of pathways in the first place. The comprehensive applicability of this method will allow the description of a vast majority of different molecular classes, which will be topic of future investigations.

Other chemical dynamics simulations for CID fragmentation analysis published so far were created via licensed programs, which limits its use to a restricted group of researchers. Therefore, a guiding principle for the development of QCxMS was its open accessibility to foster scientific exchange across disciplines. Furthermore, it may support the research and teaching segment in the understanding of fragmentation pathways and the distribution of this knowledge.

## Author Contributions

All authors conceptulized the work. J. Koopman and S. Grimme developed the QCxMS software. J. Koopman did all computational calculations. R. Schnegotzki did the Orbitrap measurements. R. Schnegotzki constructed all fragmentation schemes. J. Koopman and R. Schnegotzki wrote the manuscript. R. D. Süssmuth and S. Grimme supervised the research and reviewed the manuscript.

## Conflict of interest

The authors declare no conflict of interest.

1

## Supporting information

As a service to our authors and readers, this journal provides supporting information supplied by the authors. Such materials are peer reviewed and may be re‐organized for online delivery, but are not copy‐edited or typeset. Technical support issues arising from supporting information (other than missing files) should be addressed to the authors.

Supporting Information

Supporting Information

Supporting Information

Supporting Information

## Data Availability

The data that support the findings of this study are available in the supplementary material of this article.
