# Supplementary material for: Quantum Chemistry‐based Molecular Dynamics Simulations as a Tool for the Assignment of ESI‐MS/MS Spectra of Drug Molecules
Source: Chemistry. 2022 Apr 1;28(27):e202200318. doi: 10.1002/chem.202200318 (PMC9325386; doi:10.1002/chem.202200318)
Supplement: Supplementary file 4 — Supporting Information [file CHEM-28-0-s003.pdf]

Chemistry–A European Journal

Supporting Information

Quantum Chemistry-based Molecular Dynamics Simulations as a Tool for the Assignment of ESI-MS/MS Spectra of Drug Molecules

Romina Schnegotzki, Jeroen Koopman, Stefan Grimme,* and Roderich D. Süssmuth*

1. Calculated spectra using QCxMS[1]

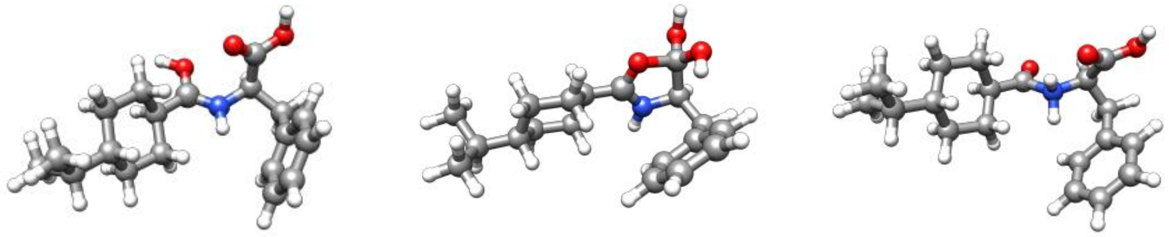

Figure S1. 3D structures of the different nateglinide protomers I (left), II (middle) and III (right).

The spectra of the three different protomers of nateglinide show the same fragments with differing intensities.

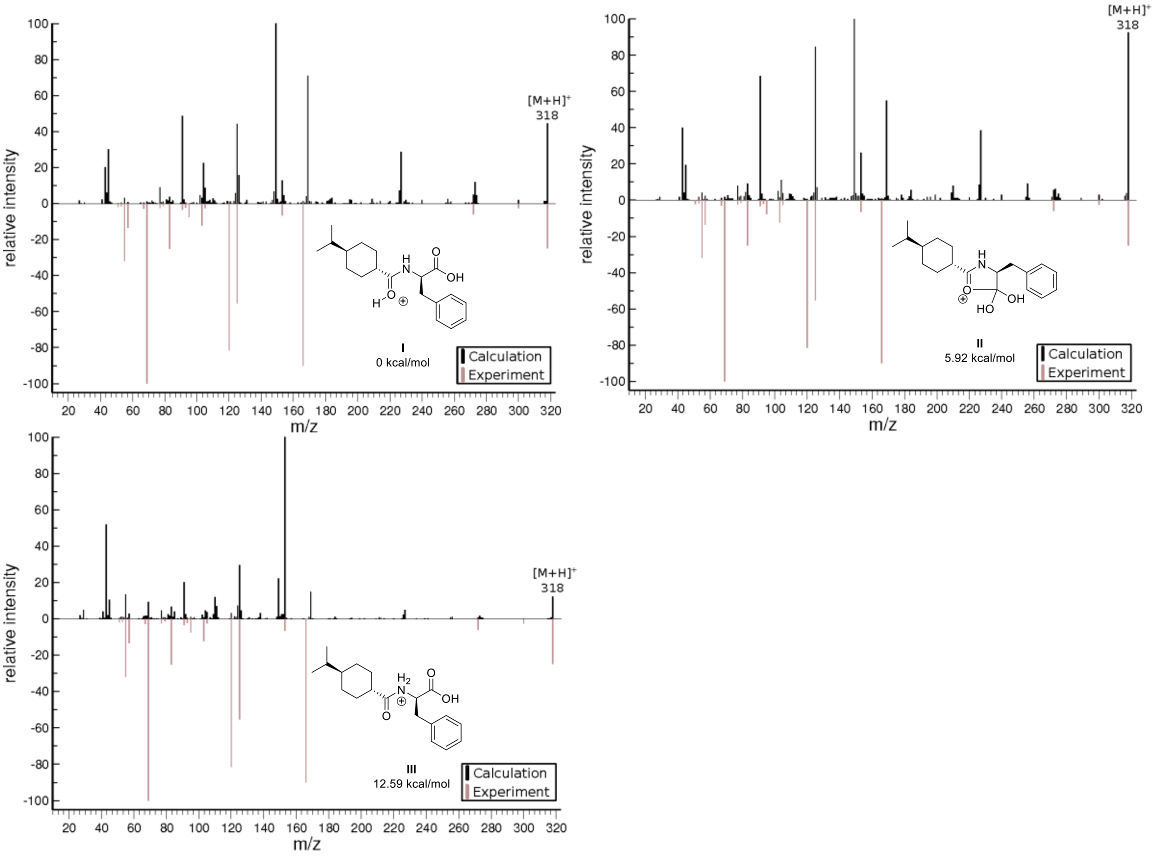

Figure S2. QCxMS simulated spectra of the three nateglinide protomers I-III.

Zopiclone

The spectra of the five different protomers of zopiclone show the same fragments with differing intensities.

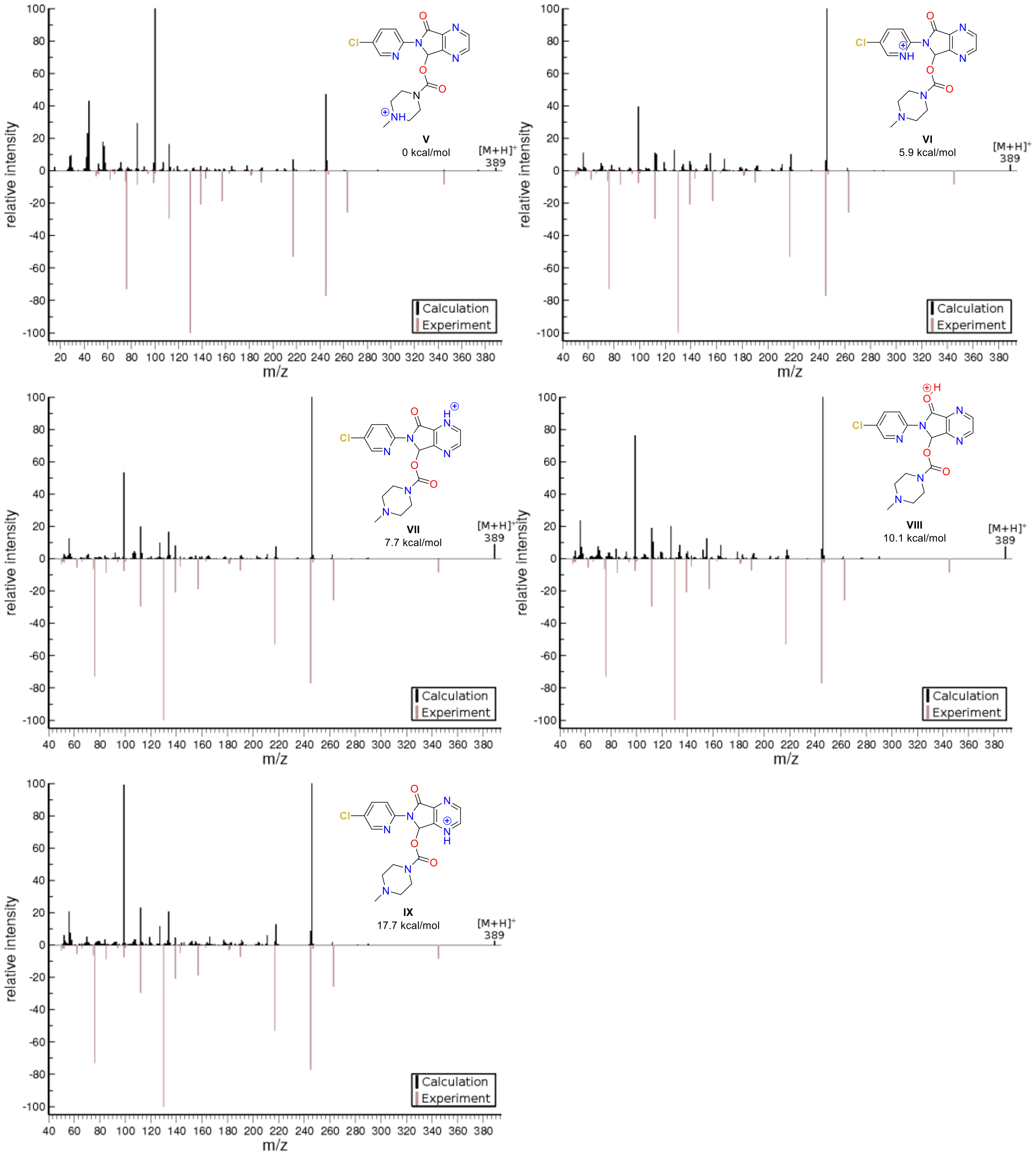

Figure S3. QCxMS simulated spectra of the five zopiclone protomers V-IX.

[1] J. Koopman, S. Grimme, *J. Am. Soc. Mass Spectrom.* **2021**, *32*, 1735.

2. Mass accuracy of the measured fragments

Table S1. Comparison of the m/z values of the measured fragment of nateglinide with the calculated values based on the sum formula.

Fragment	*m/z* of measured fragments	Calculated *m/z*	Δ mass	Sum formula
**1**	300.1962	300.1958	0.0004	$C_{19}H_{26}NO_2+$
**2**	272.2011	272.2009	0.0002	$C_{18}H_{26}NO+$
**3**	166.0862	166.0863	0.0001	$C_9H_{12}NO_2+$
**4**	153.1274	153.1274	0	$C_{10}H_{17}O+$
**5**	125.1325	125.1325	0	$C_9H_{17}+$
**6**	120.0808	120.0808	0	$C_8H_{10}N+$
**7**	105.0448	105.0335	0.0113	C_7H_5O+
**8**	103.0543	103.0543	0	C_8H_7+
**9**	95.0492	95.0492	0	C_6H_7O+
**10**	93.0700	93.0699	0.0001	C_7H_9+
**11**	91.0543	91.0543	0	C_7H_7+
**12**	83.0857	83.0855	0.0002	$C_6H_{11}+$
**13**	79.0544	79.0542	0.0002	C_6H_7+
**14**	77.0387	77.0386	0.0001	C_6H_5+
**15**	69.0700	69.0699	0.0001	C_5H_9+
**16**	67.0544	67.0542	0.0002	C_5H_7+
**17**	57.0700	57.0699	0.0001	C_4H_9+
**18**	55.0544	55.0542	0.0002	C_4H_7+
**19**	53.0387	53.0386	0.0001	C_4H_5+
**20**	51.0231	51.0230	0.0001	C_4H_3+

Table S2. Comparison of the m/z values of the measured fragment of nateglinide with the calculated values based on the sum formula.

Fragment	*m/z* of measured fragments	Calculated *m/z*	Δ mass	Sum formula
**21**	345.1227	345.1225	0.0002	$C_{16}H_{18}ClN_6O+$
**22**	263.0333	263.0331	0.0002	$C_{11}H_8ClN_4O_2+$
**23**	247.0384	247.0381	0.0003	$C_{11}H_8ClN_4O+$
**24**	245.0228	245.0225	0.0003	$C_{11}H_6ClN_4O+$
**25**	217.0276	217.0276	0	$C_{10}H_6ClN_4+$
**26**	190.0167	190.0167	0	$C_9H_5ClN_3+$
**27**	182.0587	182.0587	0	$C_{10}H_6ClN_4\cdot+$
**28**	181.0509	181.0509	0	$C_{10}H_5N_4+$
**29**	163.0057	163.0058	0.0001	$C_8H_4ClN_2+$
**30**	157.0163	157.0164	0.0001	$C_6H_6ClN_2O+$
**31**	143.0815	143.0815	0	$C_6H_{11}ClN_2O_2+$
**32**	139.0058	139.0058	0	$C_6H_4ClN_2+$
**34**	111.9949	111.9949	0	C_5H_3ClN+
**35**	100.0183	100.0182	0.0001	C_7H_2N+
**36**	99.0918	99.0917	0.0001	$C_5H_{11}N_2+$
**37**	94.0288	94.0287	0.0001	C_5H_4NO+
**38**	84.9841	84.9840	0.0001	C_4H_2Cl+
**39**	76.0183	76.0182	0.0001	C_5H_2N+
**40**	75.0105	75.0104	0.0001	$C_5HN\cdot+$
**41**	66.0340	66.0338	0.0002	C_4H_4N+
**42**	61.9793	61.9792	0.0001	$CHClN+$
**43**	52.0183	52.0182	0.0001	C_3H_2N+
**44**	51.0231	51.0229	0.0002	C_4H_3+
**45**	50.0152	50.0151	0.0001	$C_4H_2\cdot+$
